# Metabolomics profiles of short- and long-sleep duration: results from the UK biobank

**DOI:** 10.1192/j.eurpsy.2026.12087

**Published:** 2026-07-31

**Authors:** E. Toffol, G. Bernhardsen, S. M. Lehto

**Affiliations:** 1 University of Helsinki, Helsinki, Finland; 2 Akershus University Hospital, Lorenskog; 3 University of Oslo, Oslo, Norway

## Abstract

**Introduction:**

Short and, to a lesser extent, long sleep duration has consistently been found associated with increased risk of cardiometabolic disorders such as hypertension, diabetes, obesity and metabolic syndrome. The metabolomics signatures of different sleep patterns have not been examined to date in large populations.

**Objectives:**

This study aimed to examine metabolomics profiles related to sleep duration among over 270,000 participants in the UK Biobank study.

**Methods:**

The population for this study included altogether 272,318 participants with complete data on plasma metabolomics and self-reported sleep duration collected at the first assessment (2006-2010). The levels of 237 metabolites were compared in individuals with medium (7-8 h/day) vs. short (<7 h/day) vs. long (≥9 h/day) sleep duration, using univariable and multivariable linear regression models (adjusted by age, sex, education level and ethnicity). The False Discovery Rate was applied to account for multiple testing. This research has been conducted using the UK Biobank Resource under application number 99811.

**Results:**

In unadjusted models, individuals with short sleep duration (n=67,295, 24.7%; 202/237 metabolites, median estimate -0.011 SD) and long sleep duration (n=21,110, 7.8%; 222/237 metabolites, -0.029 SD) had significantly different profiles than those reporting 7-8 sleep h/day (n=183,913, 67.5%). Their profiles were suggestive of higher cardiometabolic risk, with more monounsaturated and saturated fatty acids, higher levels of the inflammation marker glycoprotein acetyls, more triglycerides, less HDL and LDL, but more VLDL cholesterol, free cholesterol, cholesterol esters, total lipids and phospholipids. The profiles remained substantially unchanged (short duration: 178/237 metabolites, 0.015 SD; long duration: 220/237 metabolites, -0.030 SD) after minimal sufficient adjustment. The magnitude of the associations was consistently larger among individuals who slept nine or more hours per day. The associations with short sleep duration partly attenuated to non-significance (96/237 metabolites, 0.001 SD) after full adjustment (age, sex, education level, ethnicity, BMI, physical activity, smoking status, chronotype and fasting time).

**Conclusions:**

Both short and long sleep duration are associated with metabolic profiles suggestive of increased cardiometabolic risk. The size of the effect seems to be larger for those reporting long sleep duration.

**Disclosure of Interest:**

None Declared

